# Enhancing broadly neutralising antibody suppression of HIV by immune modulation and vaccination

**DOI:** 10.3389/fimmu.2024.1478703

**Published:** 2024-11-07

**Authors:** Carla Nel, John Frater

**Affiliations:** ^1^ Peter Medawar Building for Pathogen Research, Nuffield Department of Medicine, University of Oxford, Oxford, United Kingdom; ^2^ The National Institute for Health and Care Research (NIHR) Oxford Biomedical Research Centre, Oxford, United Kingdom

**Keywords:** HIV, therapeutics, broadly neutralising antibodies, latency-reversing agents, immune modulators, vaccines, cure

## Abstract

Although HIV infection can be managed with antiretroviral drugs, there is no cure and therapy has to be taken for life. Recent successes in animal models with HIV-specific broadly neutralising antibodies (bNAbs) have led to long-term virological remission and even possible cures in some cases. This has resulted in substantial investment in human studies to explore bNAbs as a curative intervention for HIV infection. Emerging data are encouraging, but suggest that combinations of bNAbs with other immunomodulatory agents may be needed to induce and sustain long-term viral control. As a result, a number of clinical trials are currently underway exploring these combinations. If successful, the impact for the millions of people living with HIV could be substantial. Here, we review the background to the use of bNAbs in the search for an HIV cure and how different adjunctive agents might be used together to enhance their efficacy.

## 1 Introduction

Human Immunodeficiency virus (HIV) infection continues to impact the lives of millions of people despite the availability of effective antiretroviral therapy (ART, 1). ART inhibits viral replication and maintains undetectable plasma viral loads in the majority of people with HIV (PWH, 2). Although ART has improved the length and quality of life for PWH there are unresolved issues (3, 4). Oral ART formulations need to be taken daily, can be associated with side effects and may interact with other medications (5). Furthermore, HIV has a rapid mutation rate causing the emergence of drug-resistant variants if the stringent regimen is not adhered to (6). These effects may impact children to a larger degree as they are still in an early immunological developmental phase, drug metabolism may be different (7, 8), and adherence can be problematic (9, 10). This highlights the need for therapies which are long-acting or have the potential to lead to drug-free virological remission and even cure, particularly in regions with the highest prevalence and incidence of new infections, such as some sub-Saharan African countries. Proposals for an optimal target product profile for such a curative treatment include to be safe and tolerable, to maintain plasma HIV RNA levels below that at which transmission may occur (200 copies HIV RNA/ml) for at least >3 years or indefinitely, and to be effective in >90% of individuals. Additionally, the treatment should result in minimal drug resistance for effective long-term results (11).

## 2 Difficulties in developing an HIV cure

HIV is difficult to eradicate as it establishes itself as a latent viral reservoir in CD4+ T cells (among other cells) early in infection (12). When HIV integrates into the human genome it can become a provirus that is transcriptionally silent – or ‘latent’ - preventing the presentation of HIV antigens by human leukocyte antigen (HLA) proteins (13). This prevents these cells from being identified by cytotoxic T and natural killer (NK) cells, and are therefore “hidden” from the immune system (14). ART successfully induces viral suppression but treatment cessation usually results in the return of plasma viraemia from the latent reservoir (15).

Additionally, some antiretroviral drugs may not fully penetrate certain anatomical locations known as ‘sanctuary sites’ (e.g. brain, genital tract, and gut-associated lymphoid tissues) resulting in suboptimal drug concentrations (16, 17). The varying ability of HIV drugs to penetrate certain sites depends on numerous factors such as their physicochemical properties, the rate of perfusion to the target site, protein-binding specificity and patient-specific polymorphisms for transporters and metabolic enzymes (17). Although there is little objective evidence to support on-going replication due to sub-optimal ART in these sanctuary sites, the potential adds to the complexity of eradicating the entirety of the HIV reservoir with ART alone.

Further problems are associated with the methods available for quantifying the true size of the replication-competent HIV reservoir. Current assays include, but are not limited to, the quantitative viral outgrowth assay (QVOA), proviral (total and integrated) DNA quantitation by the polymerase chain reaction (PCR) and primer/probe based digital methods [e.g. intact proviral DNA assay (IPDA, 18); Q4PCR (19, 20)]. There are drawbacks associated with each of these assays, and their variations, which prevents consistent determination of the replication-competent latent reservoir. The QVOA assay often underestimates the true reservoir size as not all cells with intact provirus are induced with a single round of activation thus the assay only gives an estimate of the minimum frequency of latently infected cells (21). Furthermore, this assay is laborious and costly. In contrast, proviral DNA PCRs greatly overestimate the replication-competent reservoir as they are unable to differentiate between defective and replication-competent provirus (22). The IPDA assay is a digital droplet PCR assay that is an improvement on the proviral DNA PCR as it is able to infer intact proviruses that lack common defects (90% of the defective proviral sequences can be identified and excluded) thereby greatly reducing the overestimation observed with the proviral DNA PCR (23). However, it has been shown that after many years on ART intact HIV proviruses can become permanently silenced which highlights the importance of developing an assay that accurately measures both provirus intactness as well as the inducibility of the reservoir in all cell types (20). The significant heterogeneity of sensitivity and specificity of currently available assays is further hindered by a lack of standardisation across laboratories (24). Additionally, these assays are generally applied to blood samples, when the bulk of the reservoir is likely to be found in tissue.

Additional difficulties in finding an HIV cure include the limitations associated with finding a translatable and ethically sound model to test potential treatments. Non-human primate models infected with Simian Immunodeficiency Virus (SIV) or Simian human immunodeficiency virus (SHIV) have been used extensively as models for human HIV infection, and proved valuable in studies using neutralising antibodies to prevent infection (25–27). In studies assessing the role of bNAbs as treatment, NHPs infected with SHIV have been seen to be more sensitive to achieving post-treatment virological control, but this has not yet been replicated in PWH (28, 29). Lessons may also be learned from vaccine prevention studies, for example in the STEP HIV clinical trial, the same Adenoviral 5 vector (containing HIV instead of SIV), that was highly effective in controlling viremia in non-human primate challenge studies (30–32), was seen to increase susceptibility to HIV when tested in healthy people at risk of developing HIV (33).

Additional complexities to studying viral cure include the lack of validated biomarkers to reliably predict HIV-infected replication-competent cells or immune correlates of protection to predict effective responses (34, 35). Therefore, to accurately test if investigational treatments are effective in preventing viral rebound and eliminating virus-infected cells for a prolonged period, PWH must undergo a period of antiretroviral treatment interruption (ATI, 36). Although there are associated risks, ATIs can be highly informative. In non-human primate preclinical trials, some animals control after ATI and some initially rebound but then undergo post-rebound viral suppression (37). Animals receiving an additional intervention, such as broadly neutralising antibodies (bnAbs) or immunomodulators (including therapeutic vaccination), have been seen to undergo post-rebound control more frequently than those receiving only ART (38).

In clinical ATI trials if viral rebound occurs, CD4+ T cell counts could drop to low levels resulting in the resurgence of opportunistic diseases (39). Thus, highly stringent trials are designed (involving regular testing for HIV viremia and CD4+ T cell count) to ensure people are placed back on ART as soon as viremia reaches a potentially dangerous level (40). There are various views on what these viral loads and CD4+ T cell count thresholds should be. Some groups only necessitate resumption of ART after one reading above 500 000 copies/mL is obtained (41, 42), whilst for others one reading above 1000 copies/mL triggers ART re-start (43–45); others require six readings >1000 copies/ml, each one week apart (46). The risks associated with prolonged viremia as a result of overly lenient restart criteria include HIV transmission to uninfected partners, expansion of the viral reservoir, immune exhaustion, and emergence of opportunistic infections (47). In contrast, setting a too stringent threshold may result in potential post-rebound controllers being missed. Thus, the optimal viral load threshold to restart ART must balance the risks associated with prolonged viremia as well as potential immune-mediated viral control that can arise after initial rebound (48). Restart criteria were defined in a collaborative meeting as a viral load >1000 copies/mL for over 4 weeks or any single reading of >100,000 copies/mL, and a CD4+ T cell count <350 cells/uL; this is due for further review in 2024 (35). These thresholds can, however, only act as guidelines, as there are few data available to define a gold standard for all ATI trials.

Participants undergoing ATI trials have been documented to have various views on the process. Some people react positively and enjoy the experience of aiding scientific research whilst others report feeling nervous and experiencing a sense of failure when viral loads increase. This highlights the importance of thorough education and psychological support during ATI trials to ensure participants fully understand the process and are aware that they have absolute authority to resume ART at any time point (49, 50).

Despite the limitations associated with the pre-clinical models and clinical trial designs mentioned above, they have provided valuable insight into HIV drug development. In this review, the current cure strategies for HIV will be discussed focusing on bNAbs used in combination with other treatments such as immune modulators, chromatin remodellers, and therapeutic vaccines.

## 3 HIV cure strategies – an overview

An effective, practical cure for HIV is yet to be discovered. Stem cell transplantation, with donor stem cells containing a CCR5 mutation (CCR5Δ32/Δ32) that prevents viral entry, has been the only successful strategy to date (51). Although this method is highly effective it has only been performed on patients requiring a transplant for another life-threatening disease such as leukaemia or lymphoma. It is too expensive and dangerous to use on every person with HIV and thus alternative strategies must be identified. Two different concepts for an HIV cure have been described: a ‘sterilizing’ cure in which HIV is eliminated from the body and a ‘functional’ cure where HIV is still present as a provirus but remains permanently suppressed without the need for any treatment. Although these terms are less widely used, different approaches might be considered if the goal is complete viral eradication versus long-term control. These strategies might be grouped into three broad categories: immunological, transcriptional regulation and genome editing. These strategies aim to either address the problem of the latent HIV reservoir directly, or enhance immune responses to eliminate circulating virus and infected cells, or both.

### 3.1 Immunological

Immunological strategies aim to achieve a functional cure by enhancing an individual’s immune responses to induce life-long protection against HIV. These interventions include therapeutic vaccines, bNAbs and immune modulators (such as toll-like receptor (TLR) agonists). Some of these strategies – such as passive infusions of bNAbs - have been successful in inducing long-term protection in some individuals. However, as of yet, any desired protective effect is temporary across most approaches, resulting in eventual viral rebound in the vast majority of trial participants.

### 3.2 Regulation of transcription

Two different HIV cure approaches utilise transcriptional regulation. The first, the “shock and kill” strategy, aims to achieve a sterilizing cure by reactivating latent HIV provirus using latency-reversing agents (LRA) such as immune modulators and chromatin remodellers and then killing newly reactive cells with enhanced immune responses brought about by increased antigen availability (52). This strategy is often combined with immunological strategies such as bNAbs, TLR agonists, Interleukin (IL) agonists, and vaccines in an attempt to enhance the natural immune responses required to eliminate the virus. Although this seems achievable in theory, so far researchers have been unable to reactivate a large enough proportion of the latent HIV reservoir to cause a significant effect (53, 54). Furthermore, it is still uncertain whether bNAbs and immune modulators are capable of enhancing long-term immune responses that are potent enough to neutralise all circulating virus and infected cells.

The other approach termed, the “Block and Lock” strategy, aims to achieve a functional cure by utilizing latency-promoting agents (LPA) to ‘permanently’ silence proviral genes and other treatments (e.g. ART/bNAbs) to neutralise residual circulating virus (55). LPAs must induce epigenetic silencing in all latently infected cells. This brings about a deep state of latency which would limit viral rebound after ATI and which is essential for this cure strategy to be effective (56). So far attempts to achieve a deep, permanent state of latency have been unsuccessful in clinical studies (57, 58). Regular dosing of LPAs has not yet been successful in silencing the entire latent reservoir thus it may be a challenge for permanent silencing to be achievable with this strategy alone.

### 3.3 “Genome editing”

A potential exciting approach for a genome editing strategy involves directly deleting proviral genomes from host cells using CRISPR/Cas9, Zinc finger nucleases or TALEN-based technologies and killing residual circulating virus with enhanced immune responses (59). In theory, this would make viral rebound impossible resulting in a sterilizing cure. Genome editing has not yet been perfected for use on humans due to potential off-target effects and has only been successfully achieved *in vitro*, in cell culture, and *in vivo* with mouse and rat models (59–61). A study investigating Zinc finger nucleases *in vitro* was able to successfully excise full-length proviruses in 30% of cells without any cytotoxicity (62). Even though this Zinc finger nuclease trial was relatively successful *in vitro*, ideal delivery vehicles still need to be developed that preferably only allow gene editing agents to enter infected cells, before this treatment can be tested on humans (55). Additional strategies employing CRISPR/Cas9 involve mutating integrated proviral HIV DNA to become replication incompetent, but with the risk of viral escape through non-homologous end joining repair of the DNA (63, 64).

An alternative approach is to use Zinc finger nucleases to induce the CCR5Δ32 mutation in CD4+ T cells, thereby altering the person’s own DNA, rendering them resistant to HIV. An example of such modified cells was made by Sangamo BioScience and have been used in human studies. In one trial the modified cells were seen to have an estimated half-life of 48 weeks and declined at a much slower rate (-1.81 cells/day) than unmodified cells (-7.25 cells/day) during ATI. Furthermore, blood HIV DNA levels declined in the majority of the participants with one person achieving undetectable HIV RNA levels (65). In a different trial most participants only experienced a slight delay in viral rebound after ATI (about 4 weeks) compared to historical controls receiving no treatment (about 2 weeks), however, 3/14 participants (of which 2 were naturally CCR5Δ32 heterozygous) experienced partial post rebound viral control (VL<1000 copies/mL) accompanied by the restoration of CD8+ T cell responses that was not seen with the other 11 participants (66). The experienced viral control was thus likely due to a combination of the modified cells preventing viral entry and CD8+ T cells killing already infected cells. Although genome editing appears to be an attractive HIV cure approach, many ethical issues first need to be addressed, especially when conducting these experiments on human embryos. In 2018, twins genetically modified using CRISPR-Cas9 (modification in CCR5 gene) to be resistant to HIV were born. This was performed unethically and illegally, and received serious backlash due to the full consequences of genetically modifying embryos (such as the modification being transferred through generations) still being unknown (67). Despite these challenges, genome editing remains a potent and promising treatment for a prospective sterilizing cure.

## 4 Broadly neutralising antibodies

Broadly neutralising antibodies (bNAbs) are antibodies targeting conserved regions of the HIV Envelope protein (Env) capable of neutralising multiple, diverse strains (68). These antibodies either target epitopes in the CD4 binding site (VRC01 and 3BNC17), V1/V2 loop (PG16), V3 loop (10-1074 and PGT121), membrane-proximal external region or gp120-gp41 proximal interface of Env (69). The first generation of bNAbs were produced using phage display and human hybridoma electrofusion (70). These bNAbs were not very effective in suppressing viremia and displayed inadequate potency and breadth causing the rapid emergence of drug-resistant strains (71). The newest generation of bNAbs were developed using single-cell antibody cloning methods (based on BCR sequencing and recombinant expression) and high throughput neutralisation assays (70, 72–75). These bNAbs showed significant increases in potency (10 – 100 fold) and breadth (2-fold) compared to the previous generation (70). In non-human primate trials, animals treated with these bNAb monotherapies showed rapid declines in plasma RNA and proviral DNA as well as significant increases in Gag-specific T-cell responses (76). Additionally, when bNAb cocktails were administered to non-human primates shortly after SHIV infection (24hrs) there was no evidence of viraemia or establishment of viral reservoirs, and although this may arguably represent highly effective post-exposure prophylaxis in this animal model (rather than treatment) it demonstrates the potential antiviral potency of bNAbs (77).

Mixed success has been achieved in clinical trials with viraemic PWH when using short-acting bNAb monotherapy as resistant viral variants rapidly emerge (78, 79). The need for broad coverage was also an issue in two studies of bNAb monotherapy with VRC01 to prevent infection of healthy people at risk of HIV (the AMP study). The investigators concluded that there was no protection versus placebo for the cohort as a whole, but there was evidence of some protection against VRC01-sensitive strains (75% efficacy, 80, 81). Administering combinations of bNAbs has proven to be much more successful for treatment – especially in the context of concurrent ART - and reduces the frequency of viral escape mutations when both bNAbs are at therapeutic levels (82). bNAbs are being optimized to further increase their potency and breadth. One such optimization is the modification of two amino acids (M428L and N434S) in the Fc region of the antibody (83). This renders the antibodies LS variants with an extended half-life due to their increased binding affinity to the neonatal Fc receptor which promotes antibody recycling instead of degradation (70, 83). This modification has been shown to increase the period to post-treatment viral rebound, in many participants (84), and may have particular value for hard-to-reach populations.

**Figure 1:**
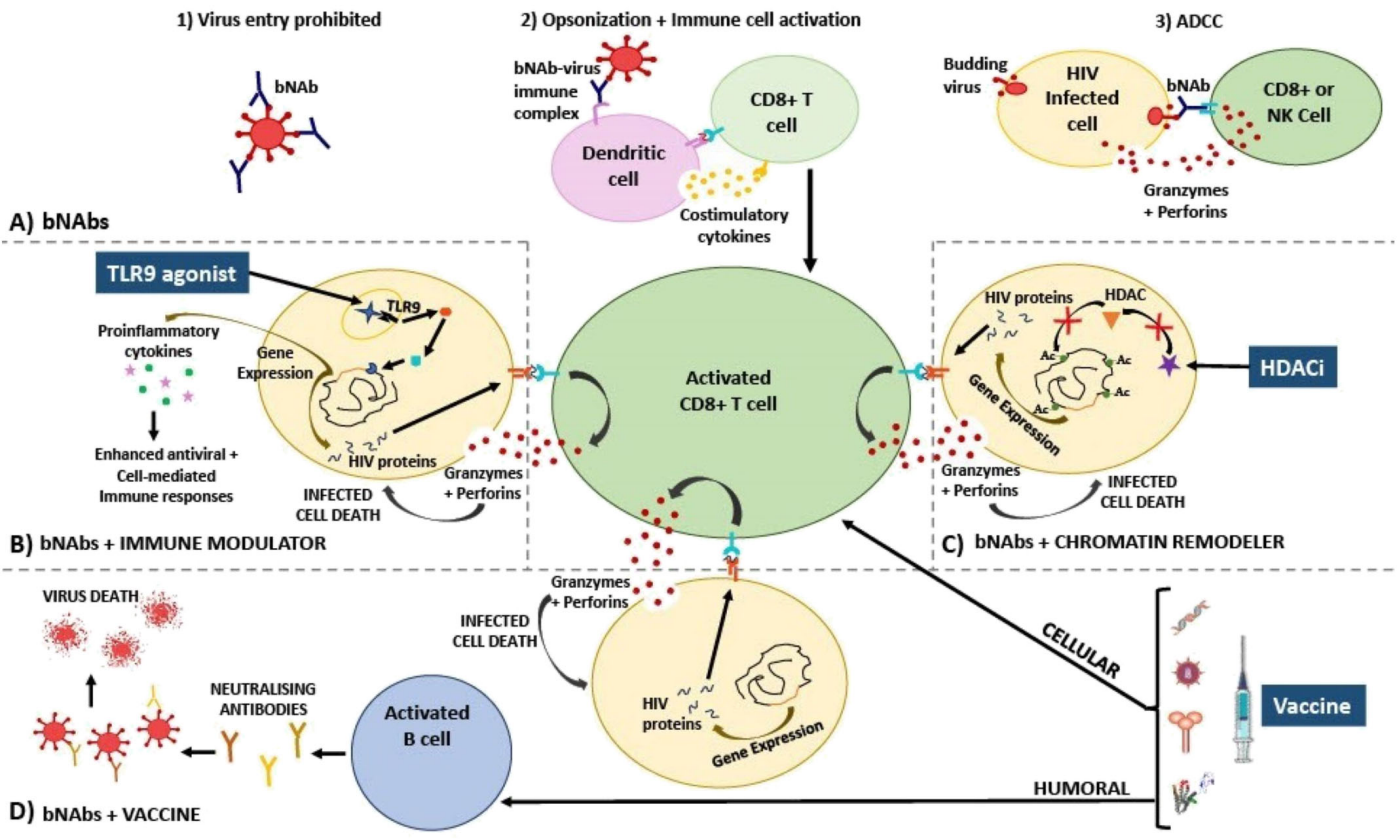
Diagram explaining the mechanism of action of HIV treatment strategies involving **(A)** bNAbs; used in combination with **(B)** immune modulators, **(C)** chromatin remodellers, and **(D)** therapeutic vaccines. **(A)** bNAbs mechanism of action has been described as inhibiting viral entry into cells via direct neutralisation (1), activating various immune cells via opsonization (2), and killing virus-infected cells via ADCC (3). **(B)** Immune modulators such as TLR9 agonists bind to receptors that activate signalling cascades in immune cells, resulting in proinflammatory cytokine gene expression, and as a result, HIV gene expression is also induced in once latently infected cells. Proinflammatory cytokines and bNAbs activate and enhance specific CD8+ T cell cytotoxic responses (release of perforin and granzyme) which are able to destroy newly activated CD4+ T cells that are presenting HIV peptides on HLA-I. **(C)** Chromatin remodellers such as HDAC inhibitors prevent the removal of acetyl groups from histone tails. As a result, nucleosomes are forced to remain further apart, keeping DNA in an open, transcriptionally active state. HIV gene expression is thus induced, reactivating the latent reservoir. Virus-infected cells are now recognized by CD8+ T cells that have been activated by bNAbs resulting in infected cell death. **(D)** HIV therapeutic vaccines should ideally amplify cellular immune responses, thereby additively enhancing CD8+ T cell activation along with bNAbs, as well as humoral (B cell) immune responses, initiating the production of neutralising antibodies towards a vast array of conserved epitopes. Additionally, these effective responses should be retained by memory T and B cells allowing sustained viral suppression when no therapy is taken, thereby bringing about a functional cure. Ac, acetyl group; ADCC, antibody-dependent cellular cytotoxicity; bNAbs, broadly neutralising antibodies; HDACi, histone deacetylase inhibitor; HIV, human immunodeficiency virus; NK, natural killer cell; TLR, toll-like receptor.

bNAbs facilitate neutralisation of circulating virus through direct binding via the Fab fragment thereby preventing viral entry into cells. They also promote the activation of other components of the immune system using their Fc fragments by allowing for the opsonization of virus by antibody-dependent cellular phagocytosis, antibody-dependent cellular cytotoxicity, and activation of the complement cascade (**Figure 1A**, 85). Additionally, it has been postulated that bNAbs induce a ‘vaccinal effect’ (86). The exact mechanism underlying this ‘vaccinal effect’ is yet to be proven and it is still uncertain which immune cells are primarily responsible. However, it is suggested that the ‘vaccinal effect’ is a process whereby antibody-virus immune complexes form and enhance antigen processing, presentation, and subsequently proliferation/activation of immune cells (86, 87). A person displaying a vaccinal effect should experience viral control off ART when bNAb levels have waned to below therapeutic levels, along with the emergence of a potent, long-lasting immune response.

CD8+ T cells are potentially key to this ‘vaccinal effect’ as seen in studies of HIV ‘elite controllers’ and demonstrated in some non-human primate trials (88). The role of CD8+ T cells in viral control has been observed in macaques receiving only ART. During ATI CD8+ T cells were shown to be responsible for maintaining a lower and stable viral setpoint in untreated macaques compared to those treated with anti-CD8+ monoclonal antibodies (89). In humans, HIV ‘elite controllers’ are seen to have increased CD8+ HIV-specific T cell responses compared to non-controllers, thus, CD8+ T cells are suspected to play a critical role in prolonged viral suppression (88). Although CD8+ T cells are thought to be a major factor contributing to viral control it is likely many other mechanisms are at play. For example, viral relapse has also been linked to the loss of antibody induction when CD4+ T cells are depleted suggesting that autologous antibody responses and T cells combine forces to control HIV, with the former helping prevent CD8+ T cell escape (90).

In a study by Nishimura et al. a significant proportion (46%) of SHIV-infected macaques that received bNAb therapy were able to maintain post-treatment viral suppression (undetectable viral loads) during ATI and after bNAb levels had waned. When anti-CD8+ monoclonal antibodies were administered to deplete CD8+ T cells viral rebound rapidly occurred in all the macaques suggesting that CD8+ T cells were responsible for maintaining viral suppression during ATI (91). This highlights that bNAbs could be part of an effective long-term treatment or cure for HIV, through CD8+ T cell induction. However, the postulated ‘vaccinal effect’ has not yet been conclusively observed in bNAb clinical trials and more studies are needed (92, 93).

The majority of evidence for bNAbs as potential therapeutics comes from studies in which they are given as passive infusions. Alternative strategies that allow for long term expression of bNAbs *in vivo* have been trialled in non-human primates with promising results. The intramuscular administration of adeno-associated virus (AAV) expressing antibody like-neutralising proteins was able to protect against virulent SIV infection 4 weeks after AAV administration with 6/9 monkey remaining uninfected after challenge (94). In a different study a monkey that received a single dose of AAV coding for an anti-SIV antibody was able to express antibody continuously for 6 years, withstanding 6 different SIV challenges of increasing infectious dose (95). Although much success has been achieved with the long-term expression of bNAbs in non-human primates, translating these effects to humans is challenging. In clinical trials testing HIV-bNAb expressing AAVs, antibody levels are not maintained at high serum titers due to multiple reasons including anti-drug antibodies and preexisting immunity to the vector. However, in some participants high bNAb concentrations have been described in the serum for up to 3 years. None of these clinical trials have tested the treatments’ ability to suppress HIV off ART (96, 97). Whether the persistent bNAb expression using AAV delivery also drives a CD8+ T cell response or ‘vaccinal effect’ (as demonstrated following bNAb passive infusions) is not known, but raises potentially interesting questions for further study.

Limitations associated with bNAbs include the prevalence of pre-existing resistance, that they may only moderately reduce the size of the latent reservoir, and that resistant viral variants readily emerge (70, 98). As bNAbs are likely only effective in people harbouring antibody-sensitive viruses this may necessitate viral screening before administration. This – and geographical viral diversity – may impact the full value of bNAbs as therapy. To date, most studies have taken place in the U.S. and Europe targeting predominantly B clade HIV, and results may not translate to other clades and other regions. Although dual bNAb therapy has proven to be an extremely promising therapy for the treatment of HIV bNAb induced viral suppression is still temporary and short lived in some participants. Whether – as for ART – a three bNAb cocktail is needed for long-term efficacy is not clear. Concerningly, in one Phase 1 trial testing the bNAbs PGDM1400, PGT121 and VRC07-523LS on ART naïve PWH, plasma HIV RNA levels were noticeably decreased after administration, however, viral rebound occurred within a median of 20 days after nadir (99). How a triple bNAb regime would sustain control in PWH on ART and then undertaking a TI is an important follow-up research question. Other adjunctive treatments such as immune modulators, chromatin remodellers or vaccines may also enhance the efficacy of bNAbs, and are the focus of the rest of the review.

## 5 Enhancing bNAbs with immune modulation

Combining bNAbs with immune modulators may enhance their potential for viral suppression and this is being tested in clinical trials. Current immune modulators being assessed include signalling agonists (such as TLR, IL, and Interferon (IFN) agonists). These signalling agonists facilitate the transcription of type I IFN and proinflammatory cytokine genes via the nuclear factor-κB (NF-κB) pathway thereby enhancing antiviral/cell-mediated immune responses (such as phagocytosis, T cell proliferation, dendritic cell (DC) maturation, and NK cell activation) targeting HIV (100). Additionally, it has been shown that activation of the NF-κB pathway via TLR agonist-receptor binding also mediates activation of the latent integrated HIV provirus (101–104). This could result in the expression and presentation of HIV proteins on the HLA-I complex of once ‘hidden’ T cells allowing them to now be recognized and killed by the immune system (**Figure 1B**).

A humanized mouse trial testing cytotoxic T-lymphocyte associated protein 4 (CTLA4), I-BET151 (immune modulators) and Vorinostat (histone deacetylase inhibitor) with the bNAbs, 3BNC117, 10-1074 and PG16 in one treatment showed that a single inducer along with the bNAbs had no significant effect on viral rebound but that a combination of all the inducers with bNAbs significantly decreased the number of rebounding animals (105). This shows how the various inducers and bNAbs might work synergistically to reduce the chances of viral rebound and supports the use of combination therapies with bNAbs as a longer-term treatment for HIV.

Similarly, many non-human primate trials have tested combinations of immune modulators and bNAbs in SHIV-infected macaques. Two trials tested the efficacy of the TLR7 agonist, Vesatolimod and bNAb, PGT121. The first trial by Borducchi et al., initiated the ART and TLR7-bNAb combination treatment early, during acute SHIV infection (7 days post-SHIV infection, 106). At the end of the trial, 55% of the animals rebounded (at a median of 112 days vs 21 days in control) and no induction of CD8+ T cell responses were seen in any of the animals. The absence of CD8+ T cell responses was potentially due to low antigen availability because of early ART treatment intervention. In all animals that maintained viral suppression, SHIV RNA remained undetectable after CD8+ T cell depletion suggesting this combination potentially eliminated the viral reservoir (106). Although this trial yielded success in maintaining viral suppression in almost half of the animals, initiating treatment very early in infection limits the size and diversity of the viral reservoir and therefore does not resemble what is feasible in the majority of people living with HIV. The trial by Moldt et al. translates more readily into a clinical setting as ART was initiated during chronic infection (a year after SHIV infection, 107). In this study, the TLR7-bNAb combination treatment was initiated 2.5 years after initiation of ART. After ATI only 50% of the combination treatment animals rebounded compared to 100% in the placebo group. Similarly to Borducchi’s study, 71% of the animals that maintained viral suppression remained aviremic after CD8 + T and NK cell depletion, suggesting that the reservoir may have also been eradicated in these animals (107). These non-human primate trials show that the TLR7 agonist and PGT121 combination suppresses viral rebound in some SHIV-infected macaques but that viral suppression is not solely due to CD8+ T cells and the vaccinal effect, but also reservoir eradication.

There are many clinical trials testing combinations of bNAbs with immune modulators in the pipeline. Many are still ongoing (NCT05281510, NCT05245292, NCT04340596, 108–110), whilst one has presented provisional results at a conference (NCT03588715 - BEAT-2, 111) and the other is completed and results have been published (NCT03837756 – TITAN Trial, 112). In the TITAN trial, the TLR9 agonist, Lefitolimod, and bNAbs (10-1074 and 3BNC117) were administered in combination and individually to PWH during ATI (28). The time to viral rebound was 0.5, 12.5, and 9.5 weeks longer than the placebo in the Lefitolimod only, bNAbs only, and Lefitolimod plus bNAbs groups, respectively. In general, HIV-specific CD8+ T cells were significantly increased in patients with higher viral loads but non-significantly increased in patients with lower viral loads suggesting that the increase was not due to the vaccinal effect but rather an increase in antigen availability. Furthermore, there was no change seen in Gag-specific CD8+ T cell responses or Gag-induced cytokine release. The authors proposed that the combination treatments’ inability to broadly stimulate cellular immunity was due to low amounts of antigen available at bNAb administration as the interventions were administered before ATI was induced. This suggests that administering immunostimulatory molecules in the same location and time where antigen is available may be important. This study showed that the addition of Lefitolimod added no clinical or immunological benefit to participants during ATI, and that the co-administration of Lefitolimod with bNAbs acted antagonistically leading to a significantly faster decline in bNAb serum concentrations compared to bNAbs alone (28). In the BEAT-2 trial where the immune modulator peginterferon alfa-2b and bNAbs (3BNC117 and 10-1074) were co-administered provisional results show that 86% of the participants maintained viral suppression for at least 26 weeks with 40% of these individuals not meeting ART restart criteria (and were still suppressing viremia) after the 38-week ATI (113). Despite the success of the treatment in inducing viral suppression, the combination did not decrease the reservoir size and there was no overall increase in HIV-specific T-cell responses.

## 6 Enhancing bNAbs with latency reversing agents

Chromatin structure plays a role in inhibiting HIV gene expression in latently infected cells. HIV proteins alter chromatin remodelling complexes as part of their natural cycle thereby controlling HIV latency and reactivation (114). Chromatin remodelling drugs such as histone deacetylase inhibitors (HDACi) can activate genes by altering the positioning of nucleosomes (115). HDACi prevent the removal of acetyl functional groups on histone lysine residues forcing the nucleosomes further apart thereby allowing DNA to remain in an open, transcriptionally active state (**Figure 1C**, 116). Additionally, the ‘Smac mimetic’ AZD5582 (a mimetic of the second mitochondrial-derived activator of caspases that antagonises apoptosis inhibitor proteins), can activate the non-canonical NF-κB signalling pathway and as a result induce HIV or SIV expression in humanized mice or non-human primates, respectively, thereby acting as a potent LRA in these models (117). AZD5582 has been tested in combination with four SHIV-specific rhesus monoclonal antibodies and the IL-15 superagonist, N-803 in SHIV-infected, ART supressed rhesus macaques (118). The trial showed that using AZD5582 in this combination resulted in a significant reduction of total and replication competent SIV DNA in lymph node derived CD4+ T cells compared to either of the treatments alone, indicating this combination successfully reduced the viral reservoir (118). In this study the bNAb-LRA combination worked additively to yield a greater effect than each intervention in isolation. Despite AZD5582 success in isolation and in combination with bNAbs in non-human primates, the drug has not yet been optimised for use in humans (119). As of yet, HDAC inhibitors are the only LRAs that have been used in combination with bNAbs in clinical trials. The HDAC inhibitors used in these clinical trials include Vorinostat and Romidepsin. Three clinical trials testing the combination treatment of bNAbs and HDAC inhibitors have been published to date.

Two of these trials tested the efficacy of the bNAb, 3BNC117, and HDACi, Romidepsin, in PWH during ATI. The trials differed in that one administered the treatment along with ARVs to people who had never initiated ARV therapy before (NCT03041012 – eCLEAR, 120, 121), whereas the other administered the treatment with ARVs to people who were virologically suppressed and taking ARVs for at least 18 months (NCT02850016 – ROADMAP, 92, 122). The eCLEAR trial reported increased elimination of plasma viruses and infected cells after treatment. Additionally, 80% of participants with pre-ART 3BNC117 sensitive viruses exhibited ART-free virological control for at least 12 weeks. The combination treatment also enhanced HIV-Gag-specific CD8+ T cell immunity (measured by the AIM assay) compared to ART alone. However, the combination of Romidepsin and 3BNC117 did not enhance clearance of the viral reservoir and maintain viral control more than 3BNC117 on its own. Therefore, Romidepsin exhibited no immunostimulatory or latency-reversing additive effect. In contrast, the ROADMAP trial yielded disappointing results with the same combination treatment. The combination treatment (Romidepsin and 3BNC117) did not significantly reduce HIV DNA or delay viral rebound when compared to Romidepsin alone (median time to viral rebound was 18 days and 28 days in the combination and Romidepsin-only groups, respectively). Additionally, no change in the size of the latent reservoir and no enhancement in HIV-specific cellular immunity was observed in either of the groups. The difference in success observed between the two trials may be due to the eCLEAR trial containing participants who were ART naïve at trial commencement. The immune responses induced by initial antigen availability could have been enhanced during treatment and throughout ATI as opposed to participants in ROADMAP where antigen, and as a result, likely immune responses, had been absent for over a year. Nevertheless, regardless of when ART was initiated, both trials showed that the combination of Romidepsin and 3BNC117 was no more effective in preventing viral rebound, decreasing the latent viral reservoir, and increasing HIV-specific cellular immunity than bNAbs alone.

The other clinical trial (NCT03803605 - VOR-07, 123) tested the efficacy of the bNAb, VRC07-523LS, and the HDACi, Vorinostat in stimulating expression of proviral HIV from resting CD4+ T cells whilst participants remained on uninterrupted ART (124). The combination therapy was unable to reduce low-level viremia or the latent HIV reservoir suggesting that the T cells induced by the combination treatment may be incapable of eliminating small populations of persistently infected cells when viral replication is inhibited by ART. Once again, these studies question the role of HIV antigen availability in stimulating strong, HIV-specific CD8 + T cell immune responses (88, 125).

## 7 Background to therapeutic HIV vaccines as a potential

The immune responses elicited by HIV ‘elite controllers’ are key to maintaining viral suppression off ART (88) and are a model that aims to be replicated by therapeutic vaccines (126). However, vaccine development is challenging due to HIV’s vast genetic diversity and high mutation rate (127). It is extremely difficult to find immunogens that are capable of eliciting broad and robust immune responses to all HIV strains and their possible escape mutants (128, 129). Nevertheless, many HIV therapeutic vaccines have been able to induce broad and effective cellular and humoral immune responses but none have been able to reduce the viral reservoir and prevent viral rebound (93, 130–132), challenging our understanding of the key correlates of protection.

In attempts to develop a functional cure, many researchers have designed various types of vaccines against HIV. These include, but are not limited to, DNA vaccines, viral vectored vaccines, RNA vaccines, inactivated vaccines and subunit vaccines (133).

### 7.1 DNA vaccines

DNA vaccines are recombinant bacterial plasmids containing a target gene encoding the vaccine antigen. In theory, DNA vaccines should be advantageous over some other vaccine types as they can induce both cellular (T cell) and humoral (antibody) immune responses without inducing anti-vector immunity and are easier to design and manufacture (134). However, in reality, most of these vaccines are only modestly immunogenic. In attempts to enhance the immunogenicity of DNA vaccines, adjuvants such as IL-12 and IL-15 have been added to vaccine formulations and methods have been developed to improve vaccine delivery e.g. through intradermal routes or electroporation (135, 136). These enhancements have had varying effects on the immunogenicity of DNA vaccines. In a study conducted by Jacobson et al. a multi-antigen DNA therapeutic vaccine was tested that encodes the HIV genes *gag, pol, nef, tat, vif* and *env* (MAG) alone and with a plasmid encoding the IL-12 gene administered at different concentrations (50ug, 250ug, 1000ug) via intramuscular injection combined with electroporation (135). The only significant response (measured by ICS) compared to placebo was the increase in IL-2-producing CD4+ T cells from baseline to week 14 in the MAG+IL-12(50ug) group, but no significant CD8+ T cell responses were observed (135). Seeing as the greatest response was induced by the lowest concentration of IL-12, higher IL-12 concentrations may induce negative-regulatory pathways that suppress T-cell responses. Although the MAG+IL-12(50ug) vaccine combination appeared to perform better than the MAG vaccine alone, the addition of IL-12 did not increase CD8+ T cell responses.

Some success has been achieved in the HVTN 070 and 080 studies testing the DNA vaccine PENNVAX-B (PV) alone, with IL-15 (0.8mg or 2mg) or with IL-12 (1.5mg or 1mg) administered via intramuscular injection (6mg) or intramuscular injection combined with electroporation (3mg, 136). The results showed that electroporation significantly increased the number of CD4+ and CD8+ T cell vaccine responders compared to intramuscular injection, despite containing only half the dose. In the electroporation group, the addition of IL-12 insignificantly increased the number of responders however in the intramuscular group both the addition of IL-12 and IL-15 resulted in a similar number of responders compared to PV alone. This study highlights electroporation’s superior ability to enhance immunogenicity and reduce the dose of vaccine required and demonstrates IL-12s potential to further increase immunogenicity if administered via electroporation.

Another study testing a multi-HIV antigen DNA vaccine, encoding Subtype B Rev, Nef, Tat, sections of Gag and an array of Pol and Env coded CTL epitopes, in ART naïve PWH who were infected with Subtype C viruses. Despite the discrepancy between the subtype of the vaccine and virus with which the participants were infected, significantly enhanced HIV-specific CD4+ and CD8+ T cell immune responses and a slight decrease in viral load were observed in the test group compared to placebo (137). In other DNA vaccine trials, although immune responses are sometimes enhanced, effects on viral load or viral replication are rarely seen (134).

### 7.2 Viral vectored vaccines

Viral vectored vaccines are recombinant viruses, containing a target gene encoding the vaccine antigen, rendered non-pathogenic/attenuated through the removal of genes involved in virulence. Most viral vectored vaccines are replication incompetent (genes involved with replication are removed) to improve their safety profile and to prevent off-target effects. Common viral vectors used for HIV vaccines include Poxviruses (e.g. the vaccinia viral vectors, New York Vaccinia (NYVAC) and Modified Vaccinia Virus Ankara (MVA), canarypox (ALVAC) and fowlpox), Adenoviruses (e.g. Chimpanzee Adenovirus (ChAd) and Human Adenoviruses (HAd) serotypes 5, 26 and 35) and Rhabdoviruses (e.g. Vesicular Stomatitis Virus (VSV), 138). Viral vectored vaccines are advantageous in that they elicit both potent antibody responses as well as cell-mediated immune responses. They are highly immunogenic on their own (don’t require an adjuvant) and are known to induce long-lasting immune responses that often only require a single dose (139). A key disadvantage associated with these vaccines is pre-existing immunity towards the viral vector. Pre-existing immunity can greatly decrease the efficacy of the vaccine by accelerating its elimination from the body or can cause severe side effects as a result of the immune reaction to the vector (140). Pre-existing immunity is only a problem when using viral vectors that normally infect humans such as the human adenoviruses or MVA (often in older people who were vaccinated against smallpox). There is also conflicting data that suggests pre-existing immunity may arise when the same viral vector is repeatedly administered to people as part of the same or different vaccine regimen thereby potentially neutralising the effect of other essential vaccines build on the same vector platform, but this is yet to be fully proven (141, 142).

Pre-existing immunity was seen to be a major problem during the STEP HIV prophylactic vaccine trial where a Human Adenovirus type 5 vaccine was used (143). In this trial, the vaccine group was associated with increased susceptibility to HIV compared to placebo (144). As a result, when designing viral vectored vaccines for HIV, one option is to use non-human adenoviral vectors boosted with a different vector or completely different vaccine. However, non-human adenoviral vectors or vectors with a very low seroprevalence in humans are not without fault. A rare side effect (Thrombosis with Thrombocytopenia Syndrome) was observed after vaccination with AstraZeneca’s SARS-CoV-2 vaccine (ChAdOx1-S) resulting in its withdrawal from the market in the European Union (145, 146). This vaccine utilised a Chimpanzee adenoviral vector and thus caution should be taken when developing HIV vaccines with this vector in the future. Furthermore, the use of vectors with low pre-existing immunity does not necessarily make for an effective vaccine as seen with the Phase 3 Mosaico HIV trial (HPX3002/HVTN706) where a low sero-prevalent vector, Adenovirus 26, was utilized (Ad26.Mos4.HIV, 147). The study was discontinued as the vaccine was seen to be ineffective at preventing the acquisition of HIV compared to placebo (148, 149).

More recently cytomegalovirus vectors (RhCMV) expressing SIV have been designed and tested in Rhesus macaques. The first generation of these vectors was effective in eliciting long lasting, broad cellular immune responses capable of protecting against pathogenic SIV mucosal challenge (150, 151). The macaques became infected with SIV after challenge, however, over time viral replication and spread was completely arrested, resulting in progressive viral clearance until challenged protected macaques were indistinguishable from control macaques – this method of control was termed “control and clear” (150, 152). Despite the RhCMV vectored vaccines great success, these vaccines were replication competent making their safety questionable for use in humans (153). This led to the development of an attenuated SIV RhCMV vectored vaccine with improved safety and retained ability to induce effective cellular immune responses capable of protecting against intravaginal SIV challenge using the “control and clear” concept. This vaccine was able to clear SIV infection in 59% of infected macaques and of these macaques 75% were able to control a second SHIV challenge three years after the last vaccination (153). Human CMV vectored vaccines harbouring HIV have not yet been developed and tested in humans but are awaited with interest (154).

### 7.3 RNA vaccines

mRNA vaccines contain a single-stranded piece of RNA encoding the target vaccine antigen of interest. Some nucleotides are modified in the RNA strand to extend half-life, decrease backbone immunogenicity and enhance translation (155). The RNA is encapsulated in delivery molecules, like lipid nanoparticles (LNPs), to allow for efficient uptake and expression by cells (156). mRNA vaccines pose many benefits such as being highly efficacious in inducing strong immune responses, having favourable safety profiles, and being easy to design and manufacture on a large scale (157). mRNA vaccine technology is still relatively new and thus clinical trials testing HIV mRNA vaccines are yet to be completed. However, rhesus macaque data is promising with strong humoral and cellular immune responses being elicited by mRNA vaccines at relatively low doses (158–160). Two phase 1 mRNA vaccine clinical trials currently underway in HIV-uninfected adults include the HVTN302 trial (NCT05217641, 161) testing 3 different mRNA vaccines (BG505 MD39.3 mRNA, BG505 MD39.3 gp151 mRNA or BG505 MD39.3 gp151 CD4KO mRNA) and the IAVIG002 trial (NCT05001373, 162) testing the eOD-GT8 60mer mRNA Vaccine (mRNA-1644) and Core-g28v2 60mer mRNA Vaccine (mRNA-1644v2-Core).

### 7.4 Inactivated vaccines

Inactivated vaccines are composed of whole virus that has been inactivated or killed via heat, chemicals or radiation - with formaldehyde and beta-propiolactone being the most common method for human vaccines (163). Very few inactivated vaccines have been designed for HIV to date. This is due to potential concerns of incomplete inactivation, technical challenges involved with vaccine manufacture and the known poorer immunogenicity associated with inactivated vaccines (164). However, inactivated vaccines are beneficial in their ability to present multiple antigens in their native conformation to the immune system which aids in the production of broadly neutralising antibodies (164). One intriguing inactivated HIV vaccine (SAV001) has been developed and tested in a clinical trial with PWH on ART (NCT01546818, 165, 166). SAV001 is a clade B HIV-1 virus genetically modified to be less virulent but more replication efficient (nef and vpu genes deleted) than the original virus, that is “killed” by inactivation with aldrithiol-2 and γ-irradiation. This vaccine was shown to be safe and effective in generating antibodies towards HIV p24, p17, gp120, and gp41 proteins as well as trimeric HIV Env glycoproteins on infected cells surfaces. Additionally, about 50% of participants showed an enhancement of broadly neutralising antibodies from baseline against HIV-1 B (tier I and II), D, and A subtypes using a luciferase-based assay to measure neutralisation activity. However, as ATI was not performed it is unknown whether the antibodies elicited by the vaccine are protective enough and capable of preventing viral replication.

### 7.5 Subunit vaccines

Subunit vaccines contain purified or recombinant immunogenic antigens of the virus that are responsible for inducing protective immune responses. Subunit vaccines only contain isolated parts of the virus making them a safe, although potentially less immunogenic, option. This makes adjuvants an essential component of the subunit vaccine formulation (167). Optimal recombinant immunogens that mimic the native conformation of the viral envelope spike of env are yet to be designed resulting mostly in a subpar elicitation of broadly neutralising antibodies by these vaccines (168–170). HIV-1 gp160 and gag p24 subunit vaccines have both been seen to induce HIV-specific immune responses and improve CD4+ T cell counts (171, 172). Most success has, however, been found when combining multiple HIV peptides into a singular recombinant subunit vaccine. An example of such a therapeutic vaccine is Vacc-4x, which contains four synthetic peptides of the HIV core protein, p24. Vacc-4x was seen to be safe, immunogenic and effective in reducing viral load (threefold reduction) when tested in a clinical trial, however, complete viral suppression was not induced (173).

### 7.6 Therapeutic vaccine combinations (clinical trials)

In attempts to maximize the benefits associated with the various vaccines discussed above, many trials use different vaccines in a combinatorial approach as part of a prime-boost vaccination strategy.

In the majority of HIV vaccine trials using different vaccine combinations, long-lasting viral suppression is not maintained during ATI. No significant effect on viral rebound was observed when using an HIVpDNA/IL-12 prime and rVSV gag boost vaccine (93). However, more promising results were seen in the AELIX-002 trial (NCT03204617, 174), which tested the efficacy of three different HIV therapeutic vaccines containing the HTI (HIVACAT) T-cell immunogen (175). The vaccine formulations tested in the trial included DNA.HTI, MVA.HTI and ChAdOx1.HTI. Of the vaccine recipients with no beneficial HLA class I controller alleles, 40% were able to continue ATI for 22 weeks as their plasma viral loads were below 10,000 copies/mL compared to only 8% of the placebo recipients. Additionally, the vaccine combination was able to induce robust, polyfunctional, and broad CD4+ and CD8+ T cell responses compared to the placebo group. Despite the vaccine immune responses being prominent and time to re-starting ART being delayed in the vaccine group, the criteria for re-start was a plasma viral load above 10,000 copies/mL, and a number of participants had detectable viral loads. This study demonstrates the promise combination vaccines might hold if administered in tandem with other treatments like LRAs and bNAbs. The majority of the vaccines described above have been shown to induce robust T-cell responses whilst LRAs [Romidepsin (120)]/immune modulators [Lefitolimod (28), peginterferon alfa-2b (113)], and dual bNAb therapies [10-1074-LS, 3BNC117-LS (82)] are thought to induce longer-term viral suppression off ART and sometimes cause reductions in the latent reservoir (69). Thus, the additive effects of these two treatments could prove to be invaluable in finding a cure or longer-term treatment for HIV.

## 8 Enhancing bNAbs with therapeutic vaccines

Neither bNAbs or vaccines alone have so far been able to consistently induce sustained viral control off ART in PWH. However, each has shown potential promise on their own. This raises the question of whether combining bNAbs with vaccination (and possibly other immune modulators) might be immunogenic enough to induce long-term viral remission. If an HIV vaccine combined with bNAbs is able to produce a strong, long-lasting, and specific cellular and humoral immune response against HIV, viral suppression could be mediated by the body’s own cells instead of ART (**Figure 1D**). This, in theory, would prevent viral rebound when no treatment is taken, bringing about a functional cure.

A non-human primate trial testing the TLR7 agonist vesatolimod, bNAb PGT121, and Ad26/MVA vaccination combination on SHIV-infected macaques supplied evidence for the potential additive effect of multiple therapeutic interventions administered together (38). Compared to the control group where 15/15 macaques rebounded, 8/12 (66%) rebounded in the PGT121 plus vesatolimod group (of which one subsequently controlled) and 6/10 (60%) rebounded in the triple combination group, of which three showed subsequent control. Overall, the triple combination treatment performed the best with 70% of the animals controlling at the end of the study compared to 41% and 33% in the Vesatolimod-bNAb and Vesatolimod-vaccine groups, respectively. This study supports the concept that vaccines and bNAbs may combine effectively to achieve viral suppression in the absence of ART.

Many clinical trials incorporating different combinations of vaccine types, immune modulators, chromatin remodellers, and bNAbs are in the pipeline. One trial has presented some provisional results at a conference (NCT04357821, 176, 177). This trial used a combination treatment comprising of the bNAbs, 10-1074 and VRC07-523LS, the TLR9 agonist, Lefitolimod, and the IL-12 adjuvanted Gag (p24) conserved element (CE)-targeted DNA prime and MVA/HIV62B boost vaccines. 70% of the combination therapy participants maintained at least partial virological control during ATI (with one participant maintaining complete viral control for over 18 months off ART) with an overall mean time to viral rebound of 15 weeks. This study shows promising results for the use of multiple combination therapies for HIV treatment, however, the study was single-armed so no results were obtained comparing the combination treatment to that of a placebo or one treatment individually. Thus, it is unknown whether the treatments induced a significant combinatorial effect or if one intervention was solely responsible for the outcomes.

The results from the ongoing clinical trials NCT04983030 (178), NCT05769569 (RV582, 179), NCT06071767 (A5374/HIV-CORE 009, 180), NCT03619278 (HIVACAR, 181), NCT06484335 (ACHIEV, 182) and AbVax are awaited with much anticipation as they are also combining multiple different LRA, bNAb, and vaccine strategies into one treatment intervention. The NCT04983030 trial will be testing the bNAbs PGT121, PGDM1400, and VRC07-523LS along with an Ad26.Mos4.HIV prime and MVA-BN-HIV boost vaccine. The RV582 trial will be testing three different vaccines (Ad26.Mos4.HIV; MVA-BN-HIV; A244d11 gp120 with a ALFQ Liposomal adjuvant) along with the bNAbs VRC07-523LS and PGDM1400LS and the superagonist IL-15 complex, N-803. The HIV-CORE 009 trial will be testing the TLR7 agonist, Vesatolimod, the bNAbs, 3BNC117-LS and 10-1074-LS and the vaccines, ChAdOx1.tHIVconsv1 + 6 and MVA.tHIVconsv3 + 4. The HIVACAR trial will be testing the efficacy of the combination treatment comprising of the LRA, Romidepsin, the bNAb, 10-1074, and a mRNA encoding HTI with TriMiXmRNAs prime and MVA.HTI boost vaccine. The ACHIEV trial will be testing the combination of the bNabs, VRC07-523LS, PGDM1400LS with a ChAdOx1.tHIVconsv1 and ChAdOx1.HIVconsv62 vaccine prime and MVA.tHIVconsv4 and A244d11 gp120/ALFQ vaccine boost. Lastly, the AbVax trial will test the ability of the combination of three vaccines (ChAdOx1.tHIVconsv1; ChAdOx1.HIVconsv62 and MVA.tHIVconsv4) and two bNAbs (10-1074-LS and 3BNC117-LS) to prevent viral rebound and elicit a vaccinal effect during ATI. The results from these trials will provide novel insights into whether combining many different HIV treatments acts synergistically to eliminate circulating and latently infected virus, antagonistically (e.g. as a result of overstimulation, immune exhaustion or drug interactions), or shows no beneficial additive effect.

## 9 Conclusion

**Figure 2:**
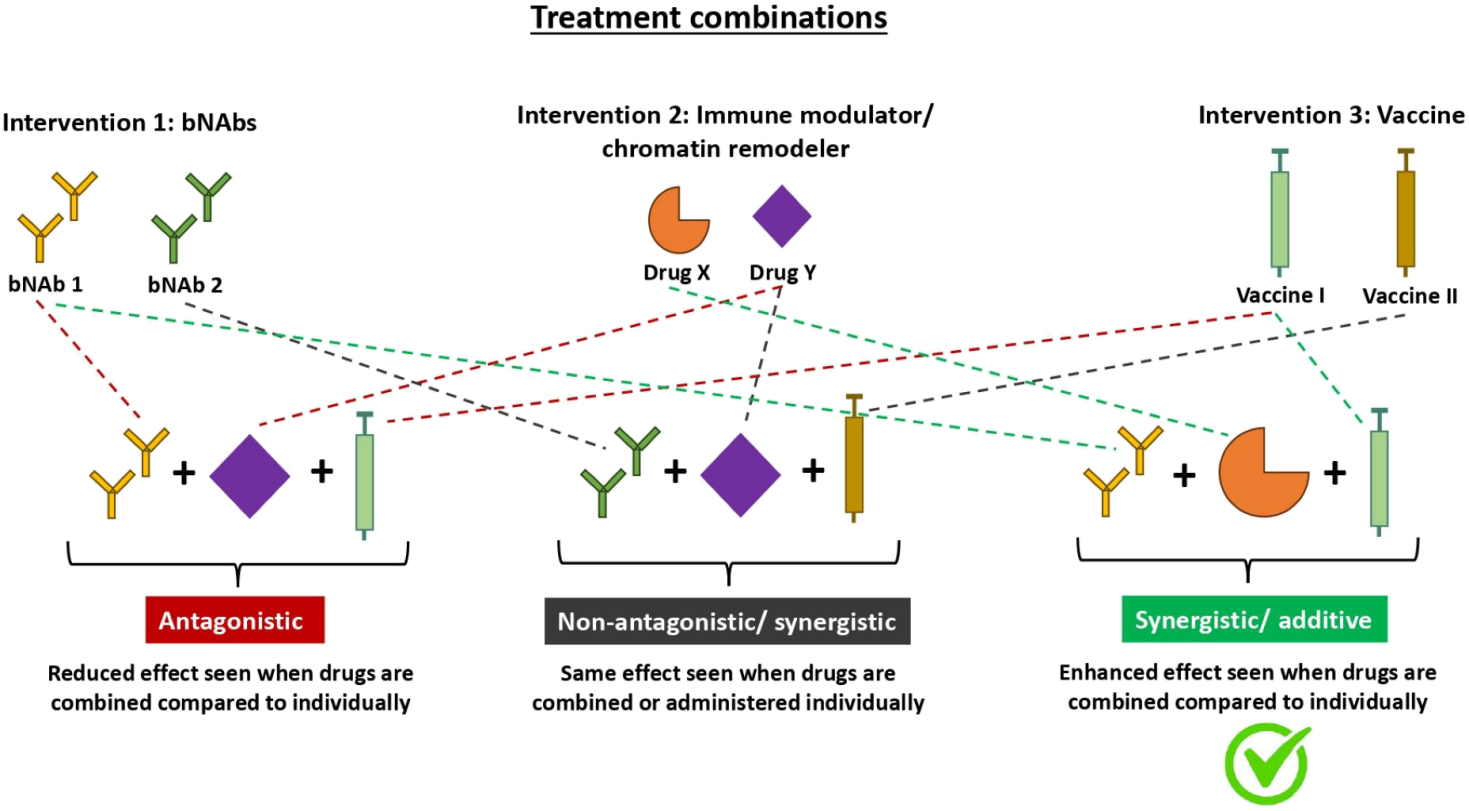
Diagram illustrating mechanisms in which different treatment combinations interact to yield various effects. Therapeutics do not always yield additive effects when used in combination. As seen with the many different types of HIV treatment combinations, the effect can either be antagonistic, additive, synergistic or neutral. Antagonistic treatment combinations (left) have a reduced effect when used in combination compared to individually. Non-antagonistic treatment combinations (middle) yield the same effect when used in combination vs individually. Whilst additive/synergistic treatment combinations (right) have an enhanced effect when used in combination compared to individually. Additive and synergistic both refer to an enhanced effect but they differ in the magnitude of enhancement. An additive interaction is when the effect of the combination treatment equals the sum of the effects of each treatment individually whereas a synergistic interaction is when the effect of the combination treatment is greater than the sum of the effects of each treatment individually. bNAbs, broadly neutralising antibodies.

Combinations of bNAbs with immune modulators, chromatin remodellers, and vaccines remain a promising strategy to achieve an effective functional or sterilizing cure for HIV. As seen by the clinical studies conducted above, many of these treatments enhance immune responses and maintain viral suppression off treatment for a reasonable period when administered alone, but their efficacy is not yet proven when administered together. Only a handful of clinical trials testing these combinations have been published with none yet available testing bNAb and vaccine combinations (**Table 1**). More research is currently on-going to determine which combinations will work synergistically or additively to amplify the responses achieved by each of the treatments alone (**Table 2**, **Figure 2**). It may very well be possible that a combination of bNAbs, immune modulators, chromatin remodellers, and vaccines will be the best strategy to achieve a cure, which is still desperately needed by many PWH. Many HIV combined bNAb-vaccine trials are currently in the pipeline or recruiting, and results from these trials over the next couple of years are greatly anticipated.

**Table 1:** Summary of the results of clinical trials involving bNAbs and other therapeutic interventions (immune modulators, chromatin remodellers and therapeutic vaccines).

Trial registration number	Trial Name	Therapeutics administered	Study Design	Viral suppression	CD8+ T cell responses
NCT03837756 (112)	TITAN (28)	TLR9: Lefitolimod bNAbs: 3BNC117, 10-1074	Phase 2a, randomised, placebo-controlled trial Participants: PWH on ART (>18 months) ATI after treatment administration	Lefitolimod/bNAb combination: antagonistic effect Viral rebound: 9.5 weeks combination vs 12.5 weeks bNAbs only	No change in Gag-specific CD8+ T cell responses/cytokine release (increase in T cells due to higher antigen availability)
NCT03041012 (121)	eCLEAR (120)	HDACi: Romidepsin bNAb: 3BNC117	Phase 2a, randomised placebo-controlled trial Participants: ART naïve PWH ATI after treatment (ART, LRA and bNAb)	Romidepsin/bNAb combination did not enhance clearance of the viral reservoir and maintain viral control more than bNAbs on their own (of the people with 3BNC117-sensitive viruses virological control was maintained for 12 week ATI)	3BNC117 (+/- Romidepsin): enhanced HIV Gag-specific immunity (AIM) and increased elimination of plasma virus and infected cells compared to ART only
NCT02850016 (122)	ROADMAP (92)	HDACi: Romidepsin bNAb: 3BNC117	Phase 2a, randomized interventional trial Participants: PWH on ART (>18 months) ATI after treatment administration	Romidepsin/3BNC117 combination: antagonistic effect Viral rebound: 18 days combination vs 28 days Romidepsin only (not clinically meaningful delay)	No significant changes in HIV-specific CD4+ and CD8+ T cell responses between baseline and pre-ATI
NCT03803605 (123)	VOR-07 (124)	HDACi: Vorinostat bNAb: VRC07-523LS	Phase 1, single-site, open-labelled trial Participants: PWH on ART (>24 months) ART maintained throughout trial	No reduction of low-level viremia or the latent HIV reservoir	(Not measured)
NCT03588715 (111)	BEAT-2 (113)	Type 1 IFN: peginterferon alfa-2b bNAbs: 3BNC117, 10-1074	Phase 1, open-labelled, non-randomised trial Participants: PWH on ART (viral load <50 copies/mL) ATI during and after treatment administration	40% of the combination therapy participants showed sustained control of viremia for at least 12 weeks after cessation of all treatment	No overall increase in HIV-specific T cell responses (AIM), increase in GAG-specific CD8+ T cell responses during ATI (ICS)
NCT04357821 (177)	None specified (176)	Vaccine: IL-12 adjuvanted Gag (p24) CE-targeted DNA prime; MVA/HIV62B boost TLR9: Lefitolimod bNAbs: 10-1074, VRC07-523LS	Phase 1/2, open-labelled, single-arm trial Participants: PWH on ART (>12 months) ATI after treatment administration	70% of participants had at least partial virological control (mean of 15 weeks) during ATI	Increased magnitude IFNγ+CE-specific CD4+ and CD8+ T cell responses in all participants

AIM, activation induced marker assay; ART, antiretroviral therapy; ATI, antiretroviral treatment interruption; bNAbs, broadly neutralising antibodies; CE, conserved element; DNA, deoxyribonucleic acid; HDACi, histone deacetylase inhibitors; HIV, human immunodeficiency virus; ICS, intracellular cytokine staining; IFN, interferon; IL, interleukin; LRA, latency reversing agent; MVA, modified vaccinia Ankara; PWH, people with HIV; TLR, toll like receptor.

**Table 2:** Summary of the current and future clinical trials involving bNAbs and other therapeutic interventions registered on clinicaltrial.gov.

Trial registration number	Trial Name	Intervention	Study Design	Primary endpoint
NCT05281510 (108)	GS-US-382-5445	TLR7: Vesatolimod bNAbs: VRC07-523LS, CAP256V2LS	Phase 2a, open-labelled, single-arm trial Participants: PWH on ART (>12 months) ATI after treatment administration	Safety (occurrence of adverse events)
NCT05245292 (109)	MCA-1031 (ES38918)	IL-15 superagonist complex: N-803 bNAbs: 3BNC117-LS, 10-1074-LS	Phase 1, open-labelled, single-arm trial Participants: PWH on ART (HIV RNA levels < 50 copies/ml) ATI after treatment administration	Safety (occurrence of adverse events) and time to viral rebound from ATI
NCT04340596 (110)	ACTG A5386	IL-15 superagonist complex: N-803 bNAbs: VRC07-523LS, 10-1074	Phase 1, randomised, open-labelled trial Participants: PWH on ART (>96 weeks) ATI after treatment administration	Safety (occurrence of adverse events) and proportion of participants with plasma HIV RNA <200 copies/mL 8 weeks after interruption of ART
NCT04983030 (178)	None specified	Vaccine: Ad26.Mos4.HIV prime; MVA-BN-HIV boost. bNAbs: PGT121, PGDM1400, VRC07-523LS	Phase 1/2a, randomised, placebo-controlled, double-blind trial Participants: PWH on ART (>48 weeks) ATI after treatment administration	Safety and tolerability; Antiviral activity measured by the percentage of participants who maintain plasma HIV RNA <1000 copies/mL; Frequency of epitope recognition measured by ELISPOT; Total IgG (including subclass) antibody titre
NCT05769569 (179)	RV582	Vaccines: Ad26.Mos4.HIV; MVA-BN-HIV; A244d11 gp120/ALFQ bNAbs: VRC07-523LS, PGDM1400LS Superagonist IL-15 complex: N-803	Phase 1, randomised, open-labelled trial Participants: PWH who initiated ART during acute infection ATI after treatment administration	Safety and time (days) from ATI to sustained viral rebound of ≥1000 copies/mL for 4 consecutive weeks
NCT03619278 (181)	HIVACAR	Vaccine: mRNA encodingHTIwithTriMiXmRNA prime; MVA.HTI boost bNAb: 10-1074 HDACi: Romidepsin	Phase 1/2a, randomised, open-labelled trial Participants: PWH on ART (>18 months) ART maintained throughout trial	Safety (Grade 3 or above severe local and systemic adverse events)
NCT06071767 (180)	A5374/HIV-CORE 009	TLR7: Vesatolimod bNAbs: 3BNC117-LS, 10-1074-LS Vaccine: ChAdOx1.tHIVconsv1 + 62; MVA.tHIVconsv3 + 4	Phase 1/2a randomised, two-arm, double-blind placebo-controlled trial Participants: PWH who initiated ART during acute infection ATI after treatment administration	Safety (occurrence of adverse events) and number of participants with viral control during ATI (VL< 1000 copies/mL at week 16)
NCT06484335 (182)	ACHIEV	Vaccines: ChAdOx1.tHIVconsv1, ChAdOx1.HIVconsv62 prime, MVA.tHIVconsv4, A244d11 gp120/ALFQ bNAbs: VRC07-523LS, PGDM1400LS	Phase 1, randomised, double-blinded, placebo-controlled trial Participants: PWH who have or will initiate ART during acute infection ATI during and after treatment administration	Safety and impact on viral load setpoint after viral rebound during ATI

Ad26, human adenovirus type 26; ART, antiretroviral therapy; ATI, antiretroviral treatment interruption; bNAbs, broadly neutralising antibodies; ChAdOx, chimpanzee adenoviral vector; ELISPOT, enzyme linked immunospot assay; HDACi, histone deacetylase inhibibtors; HIV, human immunodeficiency virus; HTI, HIVACAT T-cell immunogen; IL, interleukin; MVA, modified vaccinia Ankara; PWH, people with HIV; RNA, ribonucleic acid; TLR, toll like receptor; VL, viral load.
